# H_2_S Enhanced the Tolerance of *Malus hupehensis* to Alkaline Salt Stress through the Expression of Genes Related to Sulfur-Containing Compounds and the Cell Wall in Roots

**DOI:** 10.3390/ijms232314848

**Published:** 2022-11-27

**Authors:** Huan Li, Weiwei Zhang, Mengyuan Han, Jianfei Song, Yuansheng Ning, Hongqiang Yang

**Affiliations:** State Key Laboratory of Crop Biology, College of Horticulture Science and Engineering, Shandong Agricultural University, 61 Daizong Street, Tai’an 271018, China

**Keywords:** *Malus hupehensis*, alkaline salt, H_2_S, root, RNA-seq, cell wall, sulfur-containing compounds

## Abstract

Malus is an economically important plant that is widely cultivated worldwide, but it often encounters saline–alkali stress. The composition of saline–alkali land is a variety of salt and alkali mixed with the formation of alkaline salt. Hydrogen sulfide (H_2_S) has been reported to have positive effects on plant responses to abiotic stresses. Our previous study showed that H_2_S pretreatment alleviated the damage caused by alkaline salt stress to *Malus hupehensis* Rehd. var. *pingyiensis* Jiang (Pingyi Tiancha, PYTC) roots by regulating Na^+^/K^+^ homeostasis and oxidative stress. In this study, transcriptome analysis was used to investigate the overall mechanism through which H_2_S alleviates alkaline salt stress in PYTC roots. Simultaneously, differentially expressed genes (DEGs) were explored. Transcriptional profiling of the Control-H_2_S, Control-AS, Control-H_2_S + AS, and AS-H_2_S + AS comparison groups identified 1618, 18,652, 16,575, and 4314 DEGs, respectively. Further analysis revealed that H_2_S could alleviate alkaline salt stress by increasing the energy maintenance capacity and cell wall integrity of *M. hupehensis* roots and by enhancing the capacity for reactive oxygen species (ROS) metabolism because more upregulated genes involved in ROS metabolism and sulfur-containing compounds were identified in *M. hupehensis* roots after H_2_S pretreatment. qRT-PCR analysis of H_2_S-induced and alkaline salt-response genes showed that these genes were consistent with the RNA-seq analysis results, which indicated that H_2_S alleviation of alkaline salt stress involves the genes of the cell wall and sulfur-containing compounds in PYTC roots.

## 1. Introduction

Soil salinization seriously threatens agricultural production and plant growth. In most studies of plant salt tolerance, NaCl is usually used as a salt stress factor. However, in real saline soil, the salt mixture includes NaCl, Na_2_SO_4_, NaHCO_3_, and Na_2_CO_3_. This mixture is alkaline, which is called alkaline salt [1]. The osmotic stress, ionic stress, and high pH caused by alkaline salt stress have a more direct toxic action than neutral salt stress [2,3]. High pH impedes growth and glycolysis more significantly than NaCl stress in maize [4,5]. Revealing the salt tolerance mechanism of plants and alleviating the damage caused to plants by salt stress are of great significance for the utilization of saline–alkali land and plant cultivation. However, knowledge of the mitigation of alkaline salt stress in plants and its mechanism is still limited.

Apple is an economically important plant that is widely cultivated worldwide. Cultivated apples are planted in the soil through their rootstocks. The root is the first organ of apple to be affected by alkaline salts. Alkaline stress was reported to significantly inhibit root activity and growth, induce an increase in the superoxide anion (O_2_^−^) and H_2_O_2_ in rice and apple roots [6,7], and prevent plants from absorbing mineral nutrients [8]. The increase in glutamine synthetase activity and the soluble protein content improved the growth performance of wheat and switchgrass roots under alkaline salt stress [9,10]. Not only does the cell wall provide mechanical rigidity for plant tissues, but its physical and chemical properties can also affect cell growth. In addition, an increasing number of studies have shown that changes in the cell wall can regulate root growth under salt stress. Salt stress decreased the pectin content in cell walls, thus inhibiting soybean root growth [11]. The idea that XTH19 (xyloglucan endotransglucosylase/hydrolase) and XTH23 participate in the regulation of the development response of lateral roots to salt treatment was further confirmed in *Arabidopsis*, where BES1 functions directly upstream to control the expression levels of XTH19 and XTH23 [12]. Enhanced cell wall biosynthesis has also been shown to increase salt tolerance in broomcorn millet [13].

Hydrogen sulfide (H_2_S) is a small gaseous molecule that has physiological and signaling functions in plant tissues and cells [14]. To date, a variety of studies have demonstrated that H_2_S as a gaseous signal molecule plays a crucial role in many plant growth and abiotic stress responses [15]. Recently, there has been an important breakthrough in understanding how H_2_S is involved in the regulation of plant metabolism to enhance salt tolerance. H_2_S regulated the endogenous H_2_S metabolism, maintained the Na^+^/K^+^ balance and the oxidative stress response in cucumber [16], and also maintained ion homeostasis in rice [17] to alleviate the growth inhibition under salt stress. Moreover, evidence suggests that H_2_S interacts with nitric oxide (NO) to form a signal network, prevents K^+^ leakage and reestablishes redox balance in *Medicago sativa* [18], and reduces the Na^+^ concentration in wheat seedlings via major pathways of NSCCs and SOS1 [19]. However, most studies about the H_2_S modulation of alkaline and neutral salt stress mainly focused on the physiological mechanism of herbaceous plants [16,19]. In addition, research on fruit trees has also focused on the postharvest conservation biology of H_2_S [20], whereas limited work has been performed to explore the mechanism of H_2_S-mediated regulation of alkaline salt at the transcriptional level in fruit trees.

RNA-seq transcriptome analysis provides new ideas to reveal the mechanism of plant biological resistance to abiotic stress [21]. With this technology, the genes involved in salt and alkali tolerance have been successfully obtained from many horticultural plants, which have been proven to play a pivotal role in response to salt and alkali stress [22,23,24,25,26]. It is worth pointing out that these studies have mainly focused on the stress of simple salt or alkali conditions, and there is less information about how H_2_S alleviates alkaline salt stress in apple (*Malus*).

*Malus hupehensis* Rehd. var. *pingyiensis* Jiang (Pingyi Tiancha, PYTC), commonly used as the rootstock for apple cultivation, is resistant to waterlogging but sensitive to salinization [27]. We previously demonstrated that H_2_S pretreatment can alleviate the alkaline salt stress of PYTC roots [7]. Based on this, the current study investigated the effects of H_2_S and alkaline salt stress on gene expression profiles in *M. hupehensis* roots revealed by RNA-seq. The purposes of this study were to explore the mechanism through which H_2_S alleviates alkaline salt stress at the transcriptional level and to identify alkaline salt-responsive genes and the response to H_2_S regulation. These results will deepen the understanding of the role of H_2_S in alleviating alkaline salt stress in PYTC roots.

## 2. Results

### 2.1. Analysis of DEGs in the Roots of M. hupehensis in Response to H_2_S and Alkaline Salt Stress

**Figure 1:**
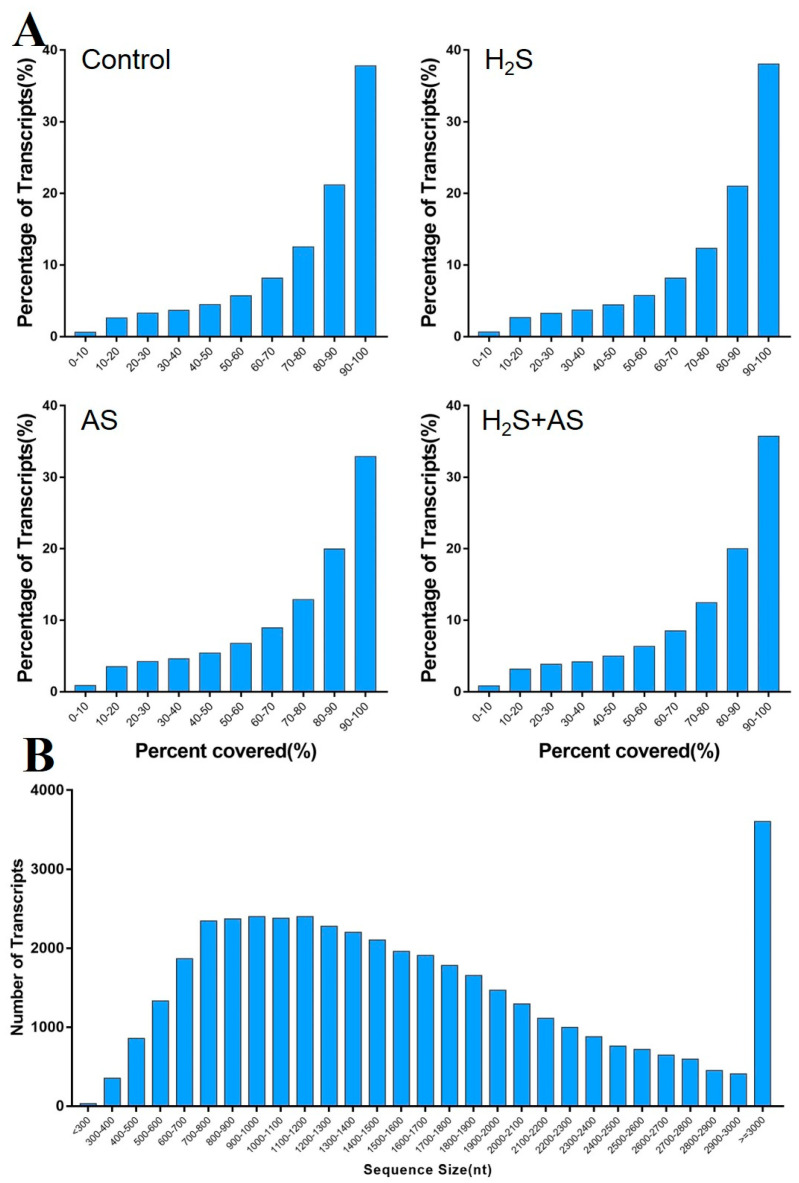
(**A**) Reads’ coverage of transcripts and (**B**) length distribution of known gene transcripts treated with H_2_S and alkaline salt stress.

Twelve cDNA libraries were collected from the Control, H_2_S, H_2_S + AS, and AS treatments with three replications and constructed using DNBSEQS Genome Analyzer deep sequencing. In total, 131.1 M (Control), 140.69 M (H_2_S), 142.64 M (H_2_S + AS), and 137.44 (AS) raw reads were obtained. After filtration, a total of 118.45 M, 125.79 M, 128.31 M, and 122.57 M clean reads were obtained from these respective libraries (Table 1). All clean reads had Phred-like quality Q20 > 96.53% and Q30 > 87.23%. As shown in Table S2, 72.23–73.85% and 66.94–69.55% of the clean reads were mapped to the *M. hupehensis* genome and gene, respectively, and only a small proportion were mapped uniquely to the genome and gene (Table S2). The transcript length and percent coverage distribution are shown in Figure 1A,B.

### 2.2. H_2_S Treatment Changed the DEG Expression Pattern of M. hupehensis Roots under Alkaline Salt Stress

**Figure 2:**
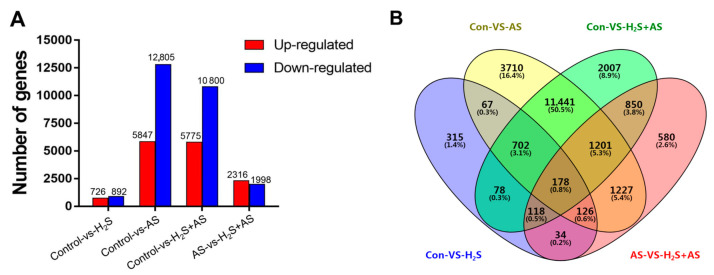
The differentially expressed genes (DEGs) of *M. hupehensis* after treatment with H_2_S and alkaline salt stress. The number of up- and downregulated DEGs between different comparison groups (**A**). Venn of the number of DEGs in different comparison groups (**B**).

The normalized FPKM was used to quantify the transcription level of the reads, thus facilitating the comparison of mRNA standards within and between genotypes. The analysis of the distribution of the gene expression level revealed that most transcripts were stimulated by H_2_S pretreatment and alkaline salt stress, and the log10 (FPKM + 1) ranged from 0 to 5 as assessed by boxplot analysis (Figure S1). This analysis indicated that the gene expression level of this study was reliable for further analysis. To improve the accuracy of the differentially expressed genes (DEGs), DEGs (Q-value < 0.001 and |log2 (fold change)| > 2) were defined as genes that were highly enriched or depleted in one treatment relative to another treatment. In the Control-H_2_S + AS, Control-AS, Control-H_2_S, and AS-H_2_S + AS groups, we identified 5775 upregulated and 10,800 downregulated genes; 5947 upregulated and 12,805 downregulated genes; 726 upregulated and 892 downregulated genes; and 2316 upregulated and 1998 downregulated genes, respectively, in *M. hupehensis* roots (Figure 2A). Venn diagram analysis showed the specificity and overlap among differentially expressed genes between different comparison groups (Figure 2B). Numerous genes overlapped in expression under H_2_S pretreatment and in response to alkaline salt stress. For example, in the Control-H_2_S + AS and Control-AS groups, 13,522 differentially expressed genes overlapped.

### 2.3. GO Term and KEGG Pathway Enrichment Analysis of DEGs between H_2_S and Alkaline Salt Stress

**Figure 3:**
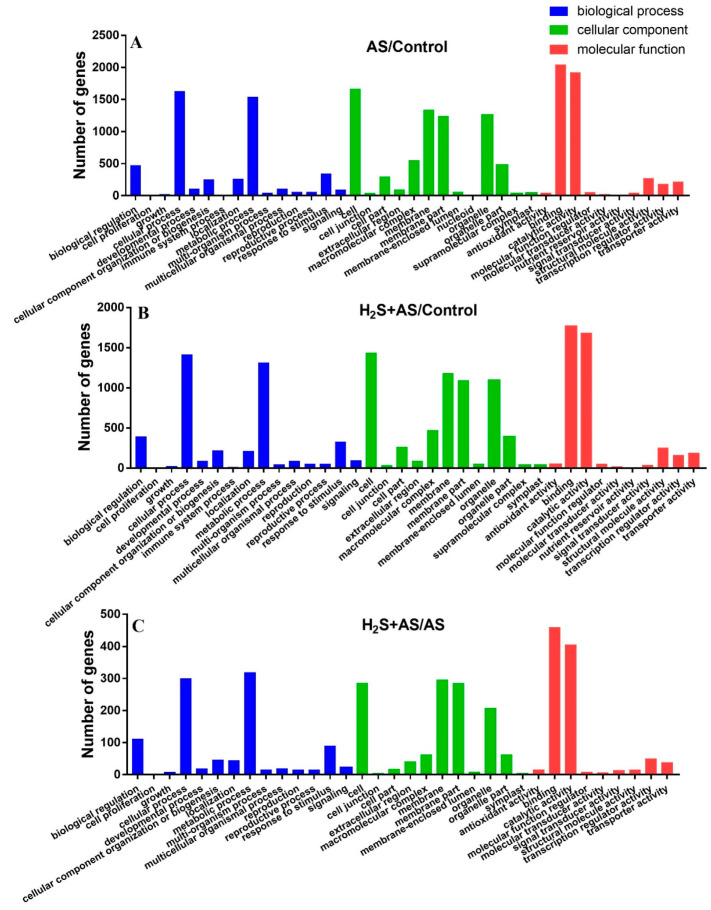
Gene ontology classification of differentially expressed genes. Blue, green, and red represent three GO ontologies: biological process, cellular component, and molecular function, respectively.

In our study, GO annotation and statistical analysis indicated that the DEGs were described as three GO ontologies (cellular component, biological process, and molecular function) with approximately 35 terms in different comparison groups (Control-H_2_S, Control-H_2_S + AS, Control-AS, and AS-H_2_S + AS). In the different comparison groups, the biological processes were similar and included “metabolic process” and “cellular process”. The “membrane”, “cell”, “membrane part”, and “organelle” were the top four highest GO terms in the cellular component. In addition, we found a significantly high abundance of molecular function categories, i.e., “binding” and “catalytic activity” (Figure 3 and Figure S2).

The functional enrichment analysis showed that the GO significant enrichment period of each comparison group (Control-H_2_S, Control-H_2_S + AS, Control-AS, and AS-H_2_S + AS) was very similar. In the biological processes of the Control-H_2_S + AS, Control-AS, and Control-H_2_S groups, the overpowering majority was “cellular process”. In the cellular component, the overpowering majority was “cell”, and “binding” was the dominant term in the molecular functions. Considering the GO biological processes, the common DEGs were significantly enriched in “cellular process” and “metabolic process”, “cell” and “cell part” of the cellular component, and “catalytic activity” and “binding” of molecular functions. Otherwise, many enriched GO terms were observed to be different in the comparison groups, in the Control-H_2_S + AS group, “hydrogen peroxide metabolic process”, “oxidoreductase complex”, and “xyloglucan: xyloglucosyl transferase activity” GO terms were identified and highly enriched. In the Control-AS group, the “ion homeostasis” and “cell wall macromolecule catabolic process” terms were identified and were highly enriched (Tables S3–S5).

**Figure 4:**
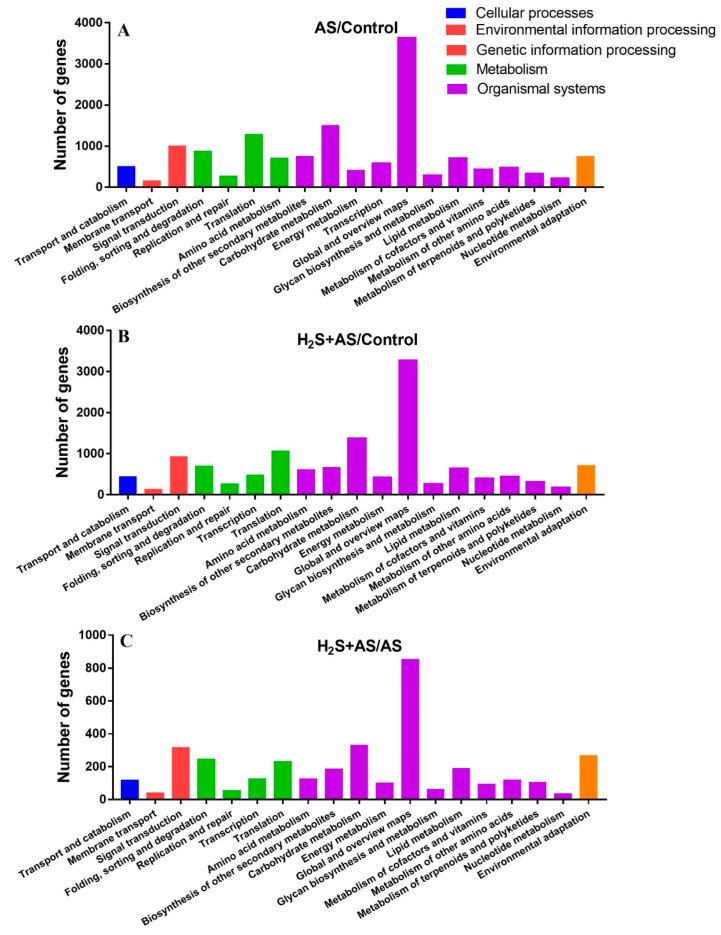
KEGG pathway of differentially expressed genes. Blue, red, green, purple, and orange represent the different KEGG pathways: cellular processes, environmental information processes, genetic information processes, metabolism, and organism systems.

We assigned 1352, 13,238, 14,820, and 3597 genes to the comparison groups (Control-H_2_S, Control-H_2_S + AS, Control-AS, and AS-H_2_S + AS) for KEGG pathway annotation, and 19 pathways were classified based on five categories, i.e., including environmental information processing, metabolism, cellular processes, genetic information processing, and organismal systems (Figure 4 and Figure S2). KEGG analysis showed that the functions of most genes were annotated into sub-branches of “signal transduction”, “translation”, and “carbohydrate metabolism”. Together, these results supported the possibility that H_2_S could alleviate alkaline salt stress by the absorption and inhibition of solutes in the root tissues of *M. hupehensis*, thus enhancing root growth.

Interestingly, the DEGs of the Control-H_2_S, Control-AS, Control-H_2_S + AS, and AS-H_2_S + AS were matched to 124, 137, 137, and 132sub-branches of the KEGG pathway, respectively. The significantly enriched KEGG pathways in H_2_S-pretreated and alkaline salt samples are shown in Tables S6–S8. Furthermore, the KEGG enrichment analysis indicated that the enriched pathways with more genes than other pathways were similar in the Control-H_2_S + AS, Control-AS, and Control-H_2_S groups, including “phenylpropanoid biosynthesis”, “MAPK signaling pathway—plant”, “plant hormone signal transduction” and “plant–pathogen interaction”. These genes were involved in multiple mechanisms that might participate in the H_2_S alleviation of alkaline salt tolerance.

### 2.4. Transcriptome Analysis and PCR Validation in Response to H_2_S and Alkaline Salt Stress

**Figure 5:**
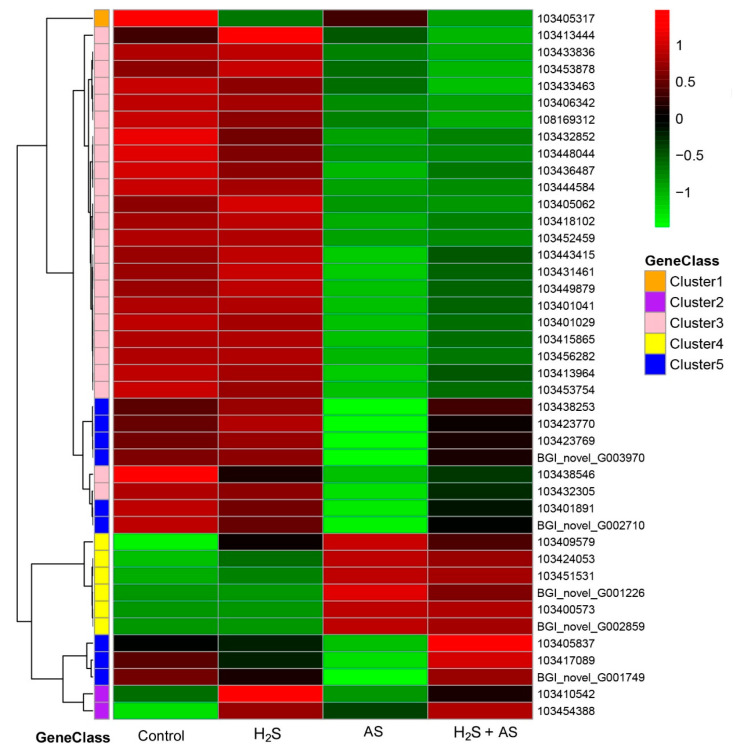
H_2_S specifically induces S-containing compound genes to alleviate alkaline salts in *M. hupehensis* roots.

**Figure 6:**
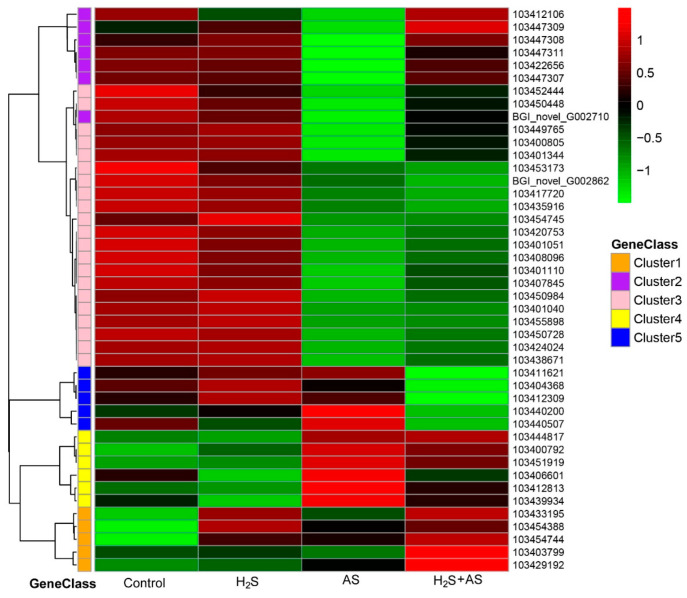
H_2_S-mediated expression of ROS-defense-related genes under alkaline salt stress in *M. hupehensis* root.

**Figure 7:**
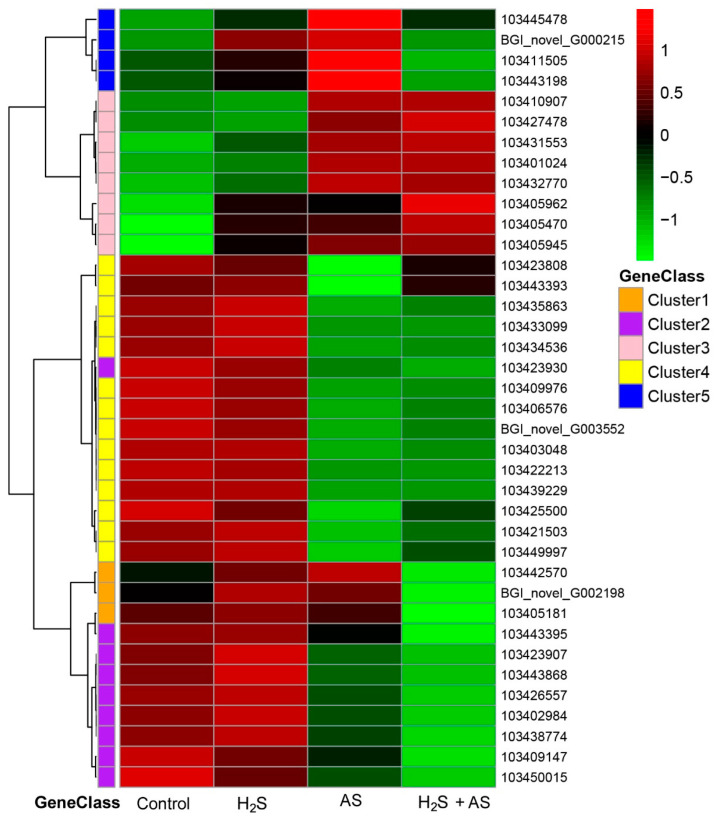
Role of cell wall metabolism–related genes in the mitigation of alkaline salt stress by H_2_S of *M. hupehensis* root.

To further explore the molecular mechanism of H_2_S pretreatment alleviating alkaline salt stress in *M. hupehensis* root, genes related to the antioxidant system and S-containing compounds, cell wall metabolism, soluble sugar, and osmoprotectants were screened from differentially expressed genes under different treatments (Tables S9–S12). Then the expression patterns of differential genes were analyzed using a heatmap (Figure 5, Figure 6 and Figure 7).

In addition, we carried out Venn diagram analysis of differentially expressed genes (DEGs) among different groups and found 126 genes that were significantly stimulated or inhibited by H_2_S and differentially expressed by alkaline salt (AS) stress induction, but there was no difference between the control group and the treatment of H_2_S + AS stress (Figure S3A). We believe that these genes are H_2_S-regulated clusters involved in alleviating alkaline salt stress. Subsequently, we compared these genes with all the identified transcription factors (TFs) for Venn diagram analysis, and a total of 15 differential TFs were identified to be involved in H_2_S-mediated AS stress relief (Figure S3B). Then, we analyzed the expression characteristics of these transcription factors using heatmaps and found that four HSF family transcription factors were highly expressed after AS stress, while, after H_2_S treatment or H_2_S + AS treatment, they were downregulated (Figure S3C,D), suggesting that these TFs may play an important role in the process of H_2_S-mediated alkaline salt stress relief.

**Figure 8:**
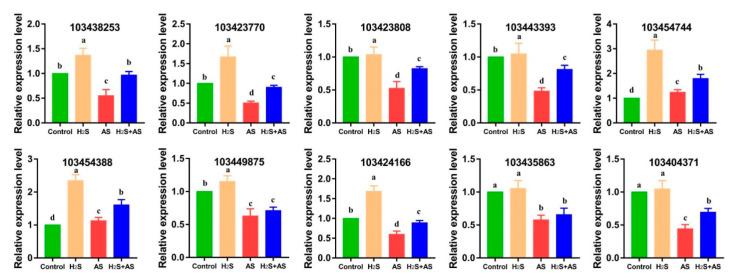
qRT-PCR analysis of DEGs in roots under different treatments. The 18S rRNA was used as a housekeeping gene for normalizing gene expression and to correct for sample-to-sample variation. The expression level of genes in the roots of plants grown under control conditions was assigned to 1. The bar represents standard deviation (SD) of the mean, different letters suggest significant differences among the treatments at *p* < 0.05.

For the examination of the expression profiles of genes that responded to H_2_S and alkaline salt treatment in *M. hupehensis* roots, we randomly selected four sulfur-containing compounds, one reactive oxygen metabolism, three cell wall metabolism, and two energy metabolism genes for PCR verification. We found that these genes correlated well with the results achieved by RNA-seq analysis (Figure 8). Therefore, the qRT-PCR results demonstrated that the RNA-seq data were reliable. Actin was used for the data normalization of the reference genes. The primers of the selected genes are shown in Table S1.

## 3. Discussion

Many studies have demonstrated that H_2_S as a signal molecule can ameliorate or alleviate the adverse response of plants to salt/alkali stress. Our previous study revealed that H_2_S can alleviate the alkaline-salt-induced toxicity on *M. hupehensis* roots through the analysis of root morphology, Na^+^/K^+^ homeostasis, ROS accumulation, and oxidative stress at the biochemical and physiological levels [7]. Nevertheless, at the transcriptome level, knowledge of the regulation mechanism through which H_2_S alleviates alkaline salt stress in *Malus hupehensis* roots remains limited. In the present analysis, we revealed many differentially expressed genes at the transcriptome level and identified signaling pathways and metabolites that might be involved in H_2_S-mediated alleviation of alkaline salt stress tolerance. In addition, it will be helpful to better understand the mechanism through which H_2_S pretreatment regulates alkaline salts stress in *Malus hupehensis* roots.

### 3.1. H_2_S-Mediated Antioxidant System and S-Containing Compounds Alleviate Alkaline Salts in M. hupehensis Roots

Salt-stress-induced oxidative stress is an effect that promotes the generation of reactive oxygen species (ROS) [28], and an increase in the levels of ROS may cause harmful oxidative stress to plant cells and organelles [29]. Evidence has demonstrated that plants can activate both enzymatic (APX, CAT, POD, and SOD) and non-enzymatic (ascorbic acid, non-protein amino acids, and glutathione) pathways to reduce ROS-induced damage [29]. It is speculated that increasing the ROS-scavenging ability of plants can significantly improve the stress resistance of plants [30,31]. Thus, under H_2_S pretreatment and alkaline salt treatment, more antioxidant and detoxification-related genes should be isolated from *M. hupehensis* roots. As expected, we identified 32 DEGs involved in ROS metabolism under salt stress. However, we identified five DEGs involved in ROS scavenging (one upregulated and four downregulated genes) and superoxide radical scavenging (one upregulated) when exogenous H_2_S was applied to apple under alkaline salt stress. We isolated two downregulated (103411621, 103412309) and one upregulated (103407845) gene involved in ROS biosynthesis of AS-H_2_S + AS, implying that the ROS biosynthesis and level might decrease upon the addition of H_2_S to the alkaline salt treatment (Figure 5 and Table S9). Our previous study showed that H_2_S pretreatment can reduce the content of ROS in the roots of *M. hupehensis* [7].

It is paramount for plants to maintain their redox status by increasing sulfur (S) metabolism and by producing S-containing compounds in response to salt-stress-induced oxidative stress. Many sulfur-containing compounds, including H_2_S, glutathione, cysteine, methionine, and thioredoxin, play essential roles in plants in stressful environments [32,33]. Sulfur metabolism is the key pathway for the biosynthesis of S-containing compounds, including H_2_S, glutathione, cysteine, methionine, and thioredoxin, playing essential roles in plants in stressful environments [32,33]. The synthesis of cysteine is the final step of sulfate reduction in plants, which is almost the only reaction for reducing sulfur for metabolism in a demand-driven manner [34,35]. Salinity can induce a higher cysteine synthesis rate [36]. As an essential amino acid, methionine not only plays a central role in the initiation of plant mRNA translation but is also a basic metabolite of plant cells and can directly or indirectly regulate a variety of cellular processes [37]. Nazar et al. discovered that glutathione plays an important role in plant metabolism and can reduce the adverse effects of salt stress on plants [38]. Our study revealed that cysteine metabolism (15 DEGs), methionine metabolism (11 DEGs), glutathione metabolism (6 DEGs), and metabolism of S-containing compounds (77 DEGs) significantly responded at the transcriptomic level to alkaline salt stress. Moreover, the glutathione S-transferase DHAR1 (103417720; 103435916), glutathione S-transferase DHAR2-like (103433463), glutamate–cysteine ligase (103438546), and glutathione synthetase (103413964) genes were significantly downregulated, similar to the result for *Catharanthus roseus*, i.e., that salinity stress decreased the glutathione content [39]. In addition, our transcriptome analysis demonstrated that the genes involved in cysteine-type endopeptidase inhibitor (103428875; 103428868; 103434431; 103438575; 103438577) that responded to the pretreatment of H_2_S before alkaline salt stress were upregulated in *M. hupehensis* roots (Figure 5 and Table S10). This indicated that H_2_S pretreatment could alleviate alkaline salt stress by upregulating the expression of S-containing compounds in *M. hupehensis* roots. This also agrees with the increased requirement for ROS scavenging, because the alkaline-salt-induced production of ROS was greater than that with the addition of H_2_S to the alkaline salt treatment of *M. hupehensis* roots [7].

### 3.2. Role of Cell Wall Metabolism–Related Genes in H_2_S Alleviating Alkaline Salt Stress

The cell wall is of vital importance to the shape of the cell and provides the necessary mechanical strength and rigidity for plant tissues to support the turgor pressure. In addition, the deposition and modification of cell wall materials play an essential role in the plant response to environmental stress [40]. Numerous studies have reported that peroxidases can be induced by salt and drought stress [41,42,43], and the peroxidase content in the cell wall of cowpea was attributed to root growth under salinity conditions [43]. Kumar et al. found that increased peroxidase activity in the cell wall could improve the resistance of transgenic tobacco to salt and drought [44]. Here, we found 4 upregulated and 28 downregulated peroxidases in *M. hupehensis* roots under alkaline salt stress. In particular, there were 11 upregulated and 2 downregulated genes involved in hydrogen peroxide catabolism, implying that the cellulose concentration may have increased in these leaves due to increased biosynthesis with H_2_S pretreatment before alkaline salt stress compared to that with alkaline salt treatment alone (Figure 6 and Table S11). A previous report suggested that H_2_O_2_ and peroxidase play an important role in the synthesis of lignin and the formation of covalent bonds between lignin and carbohydrates in the cell wall [45]. Here, we obtained two upregulated genes (103450245 and 103402291) involved in lignin metabolism with the application of H_2_S (AS-H_2_S + AS). Thus, the lignin level might be elevated with the addition of H_2_S. Collectively, this reveals that peroxidases played a role in H_2_S- and alkaline-salt-induced lignin deposition by degrading H_2_O_2_.

Many reports have demonstrated that the changes in the physical and chemical properties of the cell wall are related to cell growth [11]. The function of the cell wall as a cell growth regulator under salt stress has been investigated in detail in some studies [46,47]. Polysaccharide is the main component of cell walls, which can further reflect the density of cell walls [48], and the cell wall density and hardening activity determine the growth rate of the root system [49]. Our transcriptome analyses of *M. hupehensis* roots revealed that the expression of genes related to cell wall density and stiffening was modified by alkaline salt stress. Here, we isolated three downregulated genes (103443393, 103423930, and 103406576) involved in hydroxyproline-rich glycoprotein metabolism and two downregulated genes encoding cell wall thickening (103443882, 103444840). Xyloglucosyl transferase (eight DEGs) was significantly downregulated in alkaline salt treatment (Figure 6 and Table S11). In addition, we observed that genes encoding hydroxyproline O-galactosyltransferase HPGT1-like (103443393) and xyloglucan endotransglucosylase/hydrolase protein 32 precursor (103449997) were upregulated with the addition of H_2_S. Xyloglucan is often modified by cell wall localization enzymes and participates in cell wall modification during cell elongation [50,51]. Xyloglucan endotransglucosylase 19 (XTH19) plays an important role in the lateral root development of *Arabidopsis thaliana* in response to salt stress [12]. These results showed that changes in the expression level of cell wall density and stiffening-related genes to maintain root elongation might be necessary for H_2_S to relieve alkaline salt stress in the root system of *M. hupehensis*.

### 3.3. H_2_S Induces Soluble Sugar and Osmoprotectant-Regulation-Related Genes in Response to Alkaline Salt Stress

There is much evidence that indicates that carbohydrate and energy metabolism are critical for plant development and response to stress, including the synthesis of protective substances and ROS scavenging, for resistance against alkaline salt stress in plant roots [52,53]. Starch and sucrose metabolism determines the level of soluble sugars and affects osmotic regulation [54,55]. Among them, the increased production of soluble sugars, including proline, fructose, glucose, trehalose, and sucrose, in cells can improve the stress resistance of plants [56,57].

Here, amino sugar metabolic (seven DEGs), proline biosynthetic (three DEGs), trehalose biosynthetic (seven DEGs), glucose metabolic (nine DEGs), and starch metabolic (seven DEGs) genes significantly responded at the transcriptomic level under alkaline salt stress. More downregulated than upregulated genes related to starch metabolism and glucose metabolism were isolated from *M. hupehensis* roots under alkaline salt stress (Table S12). Similarly, these DEGs were also found with the addition of H_2_S before the alkaline salt treatment. However, the addition of H_2_S before the alkaline salt treatment could led to the disappearance of some pathways and a reduction in the number of DEGs compared to alkaline salt stress alone (Table S12). Under alkaline salt stress, genes involved in trehalose metabolism, such as trehalose-phosphate phosphatase (TPP) (103408485), trehalose-phosphatase synthase (TPS) (103446295, 103451948, 103402996), and glucose catabolism (103433794, 103408593, 103433797) were upregulated, and genes encoding sucrose-phosphatase 2 isoform X1 (103430254) and sucrose-phosphate synthase 2 (103443444) were downregulated with the transcriptome sequencing. Previous studies have shown that some TPPs’ expression levels were increased under conditions of salt stress in *A. thaliana* [58]. Similarly, salt stress upregulated the expression of TPSs in *Populus* [59]. In addition, most transgenic plants overexpressing TPS and TPP genes have great tolerance to salt stress [60,61]. In our present study, some TPP and TPSs were upregulated under salt stress, while, in the addition of H_2_S before alkaline salt treatment, these gene were not significantly different from those in the control, which suggest that these genes play an important role in salt stress and the alleviation of alkaline salt stress by H_2_S.

On the other hand, genes involved in glucose homeostasis (103424166, 103431330, and 103414773), glucose-1-phosphate adenylyltransferase (103432152 and 103405088), and fructose 1,6-bisphosphate 1-phosphatase (103416291) were significantly upregulated when H_2_S was applied to apple under alkaline salt stress (Figure 7). This result indicates that H_2_S pretreatment before alkaline salt stress induced genes involved in carbohydrate and energy metabolism, which might be different from the case when only alkaline salt stress was applied to *M. hupehensis* roots. The significant changes in genes related to primary energy metabolism and the osmoprotection of soluble sugar induced by alkaline salt stress may inhibit plant growth and development. However, the addition of H_2_S increased the gene expression abundance of these metabolic pathways and alleviated the inhibition caused by alkaline salt stress.

## 4. Methods and Materials

### 4.1. Treatment of Plant Materials

PYTC (*Malus hupehensis* Rehd. var. *pingyiensis* Jiang) seedling stratification and H_2_S pretreatment and alkaline salt treatments were performed according to our previous research methods [7]. After seedling germination, five seedlings were cultivated in black plastic bowls (diameter 11 cm and height 9 cm) that contained clean river sand and were then grown in the greenhouse under a natural photoperiod at the National Research Center for Apple Engineering and Technology Shandong Agriculture University (SDAU), China.

From the eighth day after cultivation, the seedlings in the black plastic bowls were irrigated with fresh nutrition solution containing micronutrients and macronutrients every other day until the seedlings had six to seven leaves. Thereafter, we used 0.5 mM sodium hydrosulfide (NaHS, H_2_S donor) to pretreat the seedlings, and H_2_S was dissolved in the nutrient solution and changed every 24 h. The seedlings were adapted to the nutrient solution for one day and were then treated with mixed alkaline salt (NaCl:Na_2_SO_4_:NaHCO_3_:Na_2_CO_3_ = 1:9:9:1, molar ratio; salinity 150 mM; pH 8.47–8.83). The detailed steps of the H_2_S pretreatment and alkaline salt stress treatments were performed according to our previous research methods [7]. At the end of the experiment, root samples were carefully collected and frozen in liquid N_2_ immediately and then stored at −80 °C until extraction.

Experiments were performed 3 replications per treatment with 50 plants each in completely randomized design, and the 3 replicates of each treatment were named as Control-1, Control-2, Control-3 (Control); H_2_S-1, H_2_S-2, H_2_S-3 (H_2_S treatment); AS-1, AS-2, AS-3 (AS: alkaline salt treatment); H_2_S + AS-1, H_2_S + AS-2, H_2_S + AS-3 (H_2_S and alkaline salt treatment).

### 4.2. RNA Extraction and cDNA Library Preparation for Transcriptome Sequencing

Total RNA was extracted from *M. hupehensis* roots (Control, H_2_S, H_2_S + AS, AS) with the CTAB-PBIOZOL reagent (Hangzhou Bioer Technology Co. Ltd., Hangzhou, China) using the extraction method [62]. mRNA molecules were purified from total RNA using oligo (dT)-attached magnetic beads. Subsequently, cleaved RNA fragments were generated using a random hexamer primed for first-strand cDNA, followed by second-strand cDNA synthesis. The following steps were conducted after purification: end-repair, A addition, adaptor ligation, and amplification of the cDNA fragment by PCR. Libraries were validated on an Agilent Technologies 2100 Bioanalyzer. Twelve cDNA libraries of *M. hupehensis* roots were constructed using the BGISEQ500 platform (BGI, Shenzhen, China). Three biological replicates were used for all treatments.

### 4.3. RNA-Seq Read Mapping, Assembly, and Annotation of the Transcriptome

We obtained clean reads by filtering the low-quality reads, i.e., those contaminated with adapters and those with unknown base N from the raw reads. The software TRIMmomatic (version 0.36) was used to filter the reads, and SOAPnuke (version 1.4.0) software was used for statistics. The clean reads were mapped to the reference genome sequence using Hierarchical Indexing for Spliced Alignment of Transcripts (HISAT, version 2.1.0, http://www.ccb.jhu.edu/software/hisat (accessed on 24 June 2019)) [63] and gene sequencing was conducted using Bowtie2 (version 2.5, http://bowtie-bio.sourceforge.net/Bowtie2/index.shtml (accessed on 24 June 2019)) [64]. The gene expression levels were estimated through RNA-seq using RSEM software (version1.2.8 http://deweylab.biostat.wisc.edu/rsem/rsem-calculate-expression.html (accessed on 24 June 2019)) [65]. The gene expression level of the fragments (each contig) was normalized to FPKM (per kilobase per transcript per million mapped reads). We used density distribution analysis of the filtered transcripts, stacked bar charts, and a boxplot graph to assess the expression distribution among all samples.

### 4.4. Differentially Expressed Gene Annotation and Analysis

Differentially expressed gene analysis was performed [66]. Genes that had a fold change (FC) ≥ 2 and *p*-value ≤ 0.001 were considered as differentially expressed. The Pheatmap function in R software was used for hierarchical clustering analysis of the Control, H_2_S, H_2_S + AS, and AS libraries. To determine the significant enrichment of DEGs in GO terms and KEGG pathways, the threshold was selected as the corrected *p*-value < 0.05 [67].

### 4.5. Analysis and Validation of qRT-PCR

Total RNA from the roots was extracted as described above. The DEGs from *M. hupehensis* roots were randomly chosen for qRT-PCR analysis. We designed the specific primers (Table S1) for qRT-PCR using Primer Premier 5.0 software (Premier Biosoft Int., Palo Alto, CA, USA). The qRT-PCR analysis was performed using the LightCycler^®^ 96 System (Roche Molecular Biochemicals, Lewes, UK) according to our previous research methods [62]. The 18S rRNA gene was quantified as an internal standard, and reference samples were obtained from *M. hupehensis* root control samples, which were set to 1. The 2^−ΔΔCt^ method was used to calculate differential expression. Each biological replicate sample consisted of three technical replicates.

### 4.6. Experimental Design and Statistical Analysis

Comparative analyses of the expression levels of each gene among different treatments were carried out SPSS statistical software (version 18.0, SPSS Inc., Chicago, IL, USA) (*p* < 0.05) using one-way ANOVA tests followed by the least significant difference (LSD) test.

## 5. Conclusions

**Figure 9:**
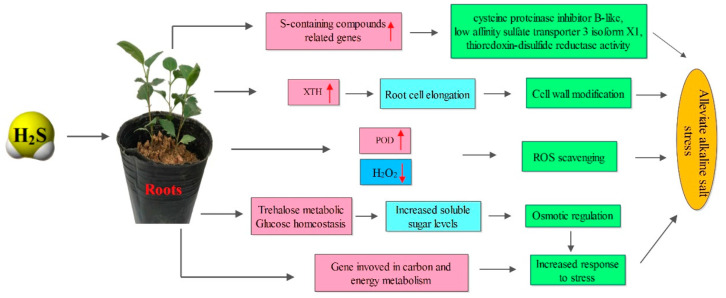
A model for the mechanisms underlying the hydrogen-sulfide-enhanced alkaline salt tolerance of *Malus hupehensis* Rehd. Hydrogen sulfide alleviates alkaline salt stress due to the activation of multifaceted defense machinery in the roots. In the roots, cell wall elongation increases because XTH gene expression is upregulated, and with the increase of POD gene expression, ROS scavenging ability was enhanced, thereby, effectively achieving osmotic regulation and detoxification of ROS.

It is a complex process to maintain or promote plant root growth under alkaline salt stress. This work presents transcriptome sequencing to analyze the effect underlying the H_2_S regulation of alkaline salt stress tolerance in *M. hupehensis* roots. The GO terms and KEGG pathway enrichment showed the response mechanism of H_2_S applied to *M. hupehensis* roots under alkaline salt stress. Investigation of the transcriptomic data suggested the candidate functional DEGs that might contribute to H_2_S regulation of alkaline salt stress tolerance in *M. hupehensis* roots. Further analysis indicated that H_2_S alleviated alkaline salt stress, including the following aspects: (a) H_2_S pretreatment resulted in higher energy maintenance capacity and cell wall integrity of *M. hupehensis* roots. (b) H_2_S pretreatment induced higher numbers of upregulated genes involved in ROS metabolism and S-containing compounds and enhanced the capacity of ROS metabolism in *M. hupehensis* roots (Figure 9). To conclude, this information on novel genes and identified DEGs in *M. hupehensis* roots will be invaluable in future studies to elucidate the specific mechanism through which H_2_S regulates alkaline salt tolerance.

## Figures and Tables

**Table 1:** Summary of the RNA-seq data collected from H_2_S and alkaline salt stress roots of *M. hupehensis*.

Sample	Raw Reads	Clean Reads (%)	Clean Bases	N (%)	Adapter (%)	Low Quality (%)	Q20 (%)	Q30 (%)
Control-1	45.09 M	40.84 M (90.58%)	6.13	97,530 (0.22%)	2,061,610 (4.57%)	2,086,720 (4.63%)	96.61%	87.44%
Control-2	42.2 M	38.23 M (90.5%)	5.73	89,922 (0.21%)	1,862,696 (4.41%)	2,061,138 (4.88%)	96.62%	87.53%
Control-3	43.77 M	39.38 M (89.98%)	5.91	93,516 (0.21%)	2,109,890 (4.82%)	2,182,808 (4.99%)	96.6%	87.47%
H_2_S-1	47.19 M	42.2 M (89.4%)	6.33	106,196 (0.23%)	2,533,522 (5.37%)	2,348,632 (4.98%)	96.61%	87.5%
H_2_S-2	46.48 M	41.74 M (89.43%)	6.26	100,562 (0.22%)	2,337,408 (5.03%)	2,302,112 (4.95%)	96.61%	87.53%
H_2_S-3	47.02 M	41.85 M (89.8%)	6.28	102,490 (0.22%)	2,621,430 (5.58%)	2,440,370 (5.19%)	96.53%	87.23%
H_2_S + AS-1	46.29 M	41.82 M (89.02%)	6.27	100,578 (0.22%)	2,083,188 (4.5%)	2,282,972 (4.93%)	96.64%	87.56%
H_2_S + AS-2	47.33 M	42.71 M (90.35%)	6.41	109,606 (0.23%)	2,246,082 (4.73%)	2,265,452 (4.79%)	96.71%	87.8%
H_2_S + AS-3	49.02 M	43.78 M (90.24%)	6.57	108,808 (0.22%)	2,832,818 (5.78%)	2,305,498 (4.7%)	96.69%	87.75%
AS-1	47.94 M	42.63 M (89.3%)	6.39	105,086 (0.22%)	2,797,352 (5.84%)	2,407,260 (5.02%)	96.61%	87.52%
AS-2	47.35 M	42.12 M (88.97%)	6.32	100,798 (0.21%)	3,043,710 (6.43%)	2,078,552 (4.39%)	96.8%	88.12%
AS-3	42.15 M	37.82 M (89.71%)	5.67	90,538 (0.21%)	2,118,994 (5.03%)	2,127,618 (5.05%)	96.55%	87.31%

AS: alkaline salt.

## Data Availability

The NCBI SRA database accession number was SRR13586165-SRR13586176.

## Supplementary Materials

The following supporting information can be downloaded at: https://www.mdpi.com/article/10.3390/ijms232314848/s1.
