# Supplementary material for: H2S Enhanced the Tolerance of Malus hupehensis to Alkaline Salt Stress through the Expression of Genes Related to Sulfur-Containing Compounds and the Cell Wall in Roots
Source: Int J Mol Sci. 2022 Nov 27;23(23):14848. doi: 10.3390/ijms232314848 (PMC9736910; doi:10.3390/ijms232314848)
Supplement: Supplementary file 1 [file ijms-23-14848-s001.zip › Supplementary Figure S1-S3 & Table S1-S2.pdf]

Supplementary material

H_2S Enhanced the Tolerance of *Malus hupehensis* to Alkaline Salt Stress through the Expression of Genes Related to Sulfur-Containing Compounds and the Cell Wall in Roots

Huan Li †, Weiwei Zhang *,†, Mengyuan Han, Jianfei Song, Yuansheng Ning and Hongqiang Yang *

State Key Laboratory of Crop Biology, College of Horticulture Science and Engineering, Shandong Agricultural University, 61 Daizong Street, Tai'an 271018, China

* Correspondence: zhangww@sdau.edu.cn (W.Z.); hqyang@sdau.edu.cn (H.Y.)

† These authors contributed equally to this work.

Table S1 Sequence of qRT-PCR primers for deferentially expressed genes

Gene ID	Gene description	Forward primer	Reverse Primer
LOC103438253	low affinity sulfate transporter 3	AATCCAATCTTCTTCCTC	ATTAGCATATCCAATACTCT
LOC103423770	uncharacterized LOC103423770	AAGTCTAGTAGTAGTAGTAGT	CTAAGGAATATCAGGATGG
LOC103423808	protein ALTERED XYLOGLUCAN 4-like	CAATATCATCACTCATCATC	AGGAGAGGAAGAATAAGA
LOC103443393	hydroxyproline O-galactosyltransferase HPGT1-like	GAACTTGATAGGATTACTG	CGCTGATAACTTCTTATG
LOC103454744	probable phospholipid hydroperoxide glutathione peroxidase	GATAAGGTGGATGTGAAT	CTAAGAGGAGAAGTTGTAG
LOC103454388	thioredoxin reductase NTRB-like	CTGAGCCTGATGAGAAGAAG	GTGGAAGCGGAAGAAGAC
LOC103449875	hexokinase-1-like	ATTATACGAGCATTACACTGAAT	CCAGAGCCATCATTAGAGT
LOC103424166	basic leucine zipper 9-like	CGTCTCACCAACCATTAC	ACAGTATCGCTCATCATCA
LOC103435863	galacturonosyltransferase 8	TGTCCATATTGAACCACTTG	AACCACTATATCGTCATCCA
LOC103404371	ferredoxin-thioredoxin reductase catalytic chain, chloroplastic	GGAGCCGTCAGATAAGTC	CCTTGATAACAACAGAAGTAACT

Table S2 Summary of clean reads and genes mapped to the reference genome and gene from H_2S and alkaline salt stress roots of *M. hupehensis*

Sample	Total Clean Reads genome	Total Mapping(%) genome	Uniquely Mapping(%) genome	Total Clean Reads gene	Total Mapping(%) gene	Uniquely Mapping(%) gene
Control-1	40.84 M	72.86 %	34.84 %	40.84 M	68.28 %	29.21 %
Control-2	38.23 M	72.97 %	34.85 %	38.23 M	68.69 %	29.13 %
Control-3	39.38 M	73.21 %	34.91 %	39.38 M	69.28 %	29.19 %
H_2S-1	42.2 M	73.24 %	35.1 %	42.2 M	68.87 %	28.77 %
H_2S-2	41.74 M	73.8 %	35.25 %	41.74 M	69.55 %	29.04 %
H_2S-3	41.85 M	72.97 %	34.82 %	41.85 M	68.22 %	28.57 %
H_2S+AS-1	41.82 M	73.48 %	34.12 %	41.82 M	68.27 %	29.91 %
H_2S+AS-2	42.71 M	73.85 %	34.44 %	42.71 M	68.89 %	29.41 %
H_2S+AS-3	43.78 M	72.89 %	34.21 %	43.78 M	67.67 %	29.03 %
AS-1	42.63 M	72.23 %	33.37 %	42.63 M	67.03 %	28.8 %
AS-2	42.12 M	72.31 %	33.89 %	42.12 M	66.94 %	28.66 %
AS-3	37.82 M	72.85 %	33.8 %	37.82 M	67.45 %	29.49 %

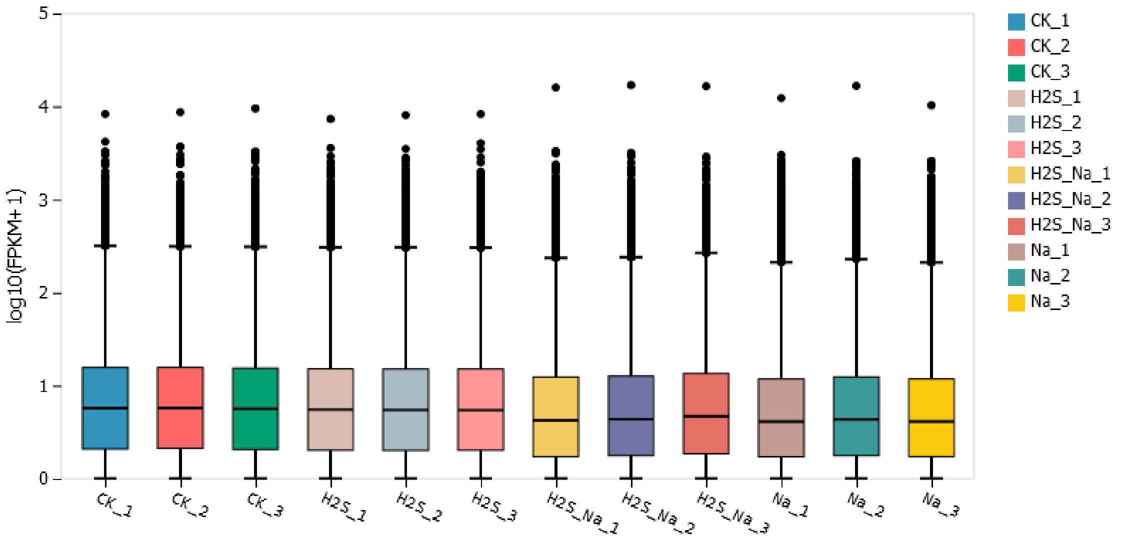

Figure S1 The box plot shows the distribution of gene expression level of each sample, and the dispersion of data distribution can be observed.

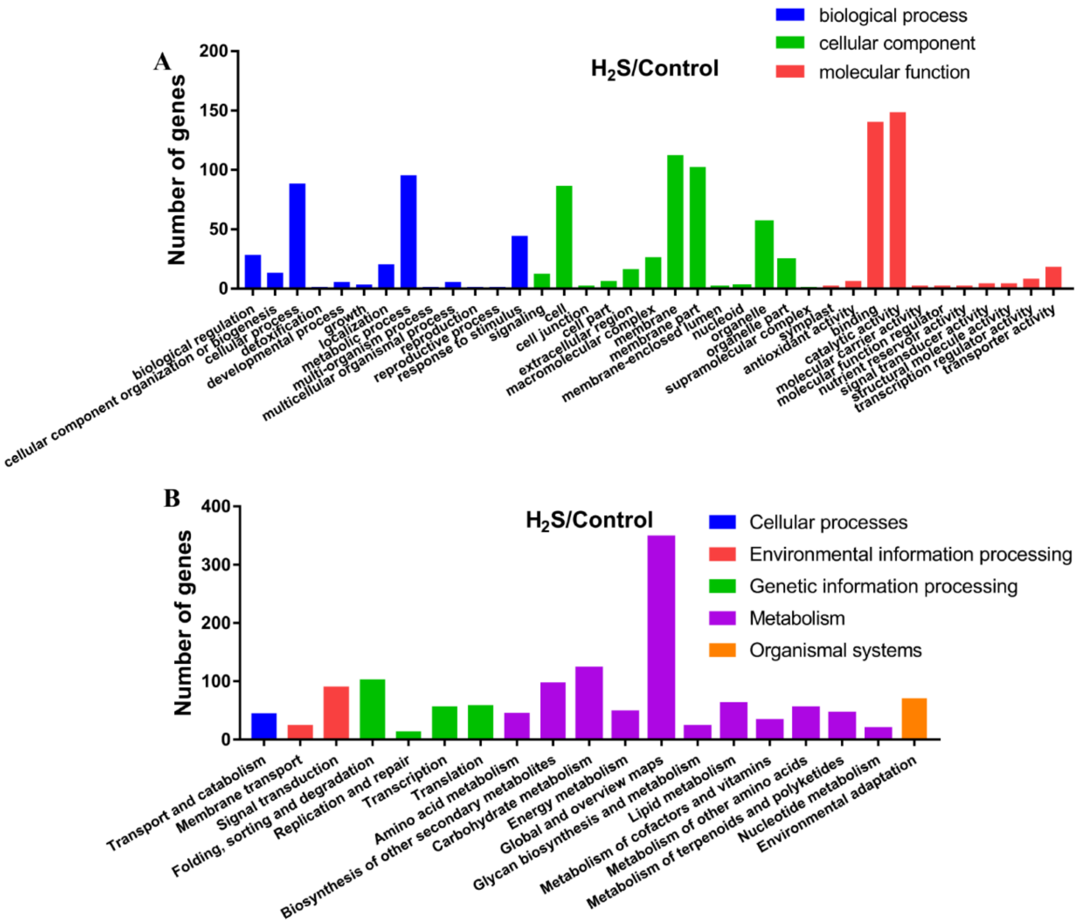

Figure S2 Gene ontology and KEGG pathway classification of differentially expressed genes in H_2S compared with control group. GO classification of DEGs. Blue, green and red represent three GO ontologies: biological process, cellular component, molecular function and, respectively **(A)**. KEGG pathway classification of DEGs. Blue, red, green, purple, and orange represent the different KEGG pathway: cellular processes, environmental information processes, genetic information processes, metabolism, and organism systems **(B)**.

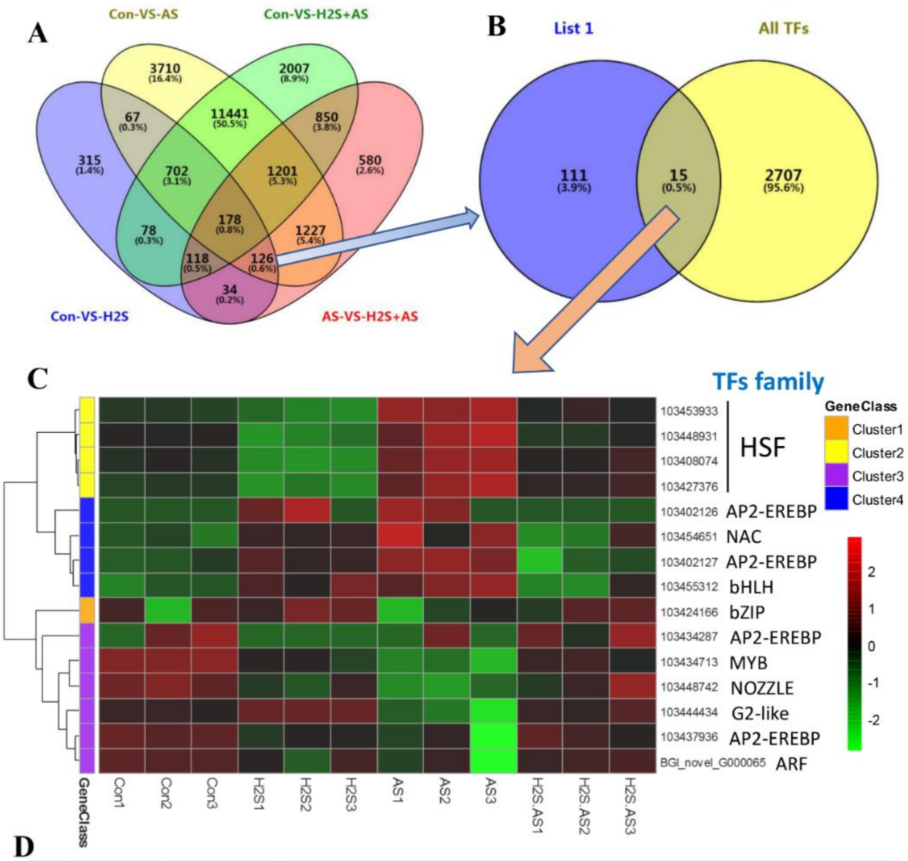

D

Gene ID	TFs family	Gene description	Protein
103453933	HSF	heat stress transcription factor A-3-like	XP_028948181.1
103448931	HSF	heat stress transcription factor A-6b-like	XP_008386414.1
103408074	HSF	heat stress transcription factor A-6b	XP_028956161.1
103427376	HSF	heat stress transcription factor A-7b-like	XP_008363653.1
103402126	AP2-EREBP	ethylene-responsive transcription factor ERF022-like	XP_008339089.1
103454651	NAC	NAC domain-containing protein 22-like	XP_008392474.1
103402127	AP2-EREBP	ethylene-responsive transcription factor ERF022-like	XP_008339090.1
103455312	bHLH	transcription factor bHLH162-like	XP_008393119.2
103424166	bZIP	basic leucine zipper 9-like	XP_028962813.1
103434287	AP2-EREBP	AP2-like ethylene-responsive transcription factor AIL1	XP_008370845.1
103434713	MYB	MYB36 protein ODORANT1-like	NP_001281023.1
103448742	NOZZLE	leucine-rich repeat extensin-like protein 5	XP_017191050.2
103444434	G2-like	probable transcription factor GLK1	XP_008381584.2
103437936	AP2-EREBP	ethylene-responsive transcription factor ERN1-like	XP_008374687.2
BGI_novel _G000065 (103431771)	ARF	auxin response factor 18-like	XP_028956236.1

Figure S3 HSF family transcription factors play an important role in the process of H_2S mediated alkaline salt stress relief. Venn of the number of DEGs in different comparison groups (A). Identification of differentially expressed key transcription factors (B). The expression patterns of differentially expressed TFs were analyzed by heatmap (C). The information of key TFs may play an important role in the process of H_2S mediated alkaline salt stress relief (D).
